# How to improve public participation in disaster risk management: A case study of Buein Zahra, a small city in Iran

**DOI:** 10.4102/jamba.v11i1.741

**Published:** 2019-08-26

**Authors:** Mojtaba Valibeigi, Majid Feshari, Fatemeh Zivari, Artemis Motamedi

**Affiliations:** 1 Department of Urban Planning, Buein Zahra Technical University, Buein Zahra, Islamic Republic of Iran; 2 Department of General Economic Affairs, Kharazmi University, Tehran, Islamic Republic of Iran; 3 Department of Civil Engineering, Buein Zahra Technical University, Buein Zahra, Islamic Republic of Iran

**Keywords:** disaster risk management, disaster risk reduction, community participation, enabling environment, organisational development, earthquake risk management

## Abstract

Identifying and providing basic solutions using a collaborative approach in earthquake-stricken cities of Iran has not yet been addressed. This article focuses on an area of practice and views disaster risk management from the point of view of volunteer groups to illustrate how the main components of disaster risk management affect the strengthening of public participation. In this article, Buein Zahra, a small city in Iran, is considered as a high-risk earthquake zone. The basic components of risk management are identified, namely public awareness, knowledge, skills, enabling environment, organisational development and social participation. An assessment of these indicators was done, and multidimensional relationships were established between them to enable an increase in the capacity for public participation. Accordingly, the results indicate that a mere increase in public awareness and knowledge, as seen today, and an improvement in enabling environment, although affecting disaster risk reduction, cannot by themselves lead to real public participation. Organisational development and strengthening of crisis coping skills are two key components to improving participation during crises in the small cities of Iran. According to the results of this study, institutional capacity and unreal political commitment have caused inefficiency of public participation in earthquake preparedness.

## Introduction

**FIGURE 1:**
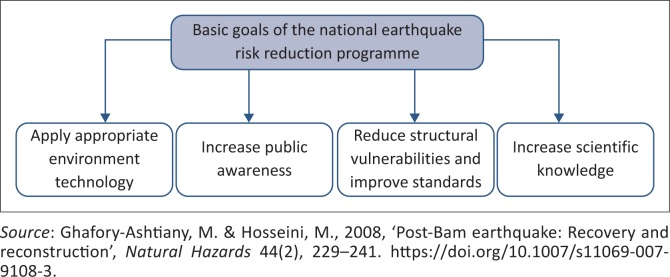
Basic goals of the national earthquake risk reduction programme.

Iran is exposed to a high level of seismic hazards throughout the country. It has become evident that a long-term vision is required to reduce the level of risk for the population (ISDR 2004; Peduzzi et al. 2009). During the last 50 years, earthquakes have killed more than 180 000 people. Many cities, including Buein Zahra (7.2 M_L_), Tabas (7.7 M_L_), Rudbar-Manjil (7.4 M_L_) and Bam (6.5 M_L_), have persistent significant damage because of high-magnitude earthquake activities. Review of the historical seismic data shows that almost all parts of the country are affected by the physical, social and economic problems associated with earthquakes (Giardini et al. 1999). According to the earthquake zoning map, 67% of the vast area of Iran is at risk of an earthquake and only 3% of the cities in Iran are in low-risk areas (ISDR 2004). The development of a national policy of disaster risk reduction (DRR) was promoted largely by scientific groups and technical interests. After the Manjil earthquake in 1999, a multilateral and interdisciplinary national earthquake risk reduction plan was developed. The plan, as shown in Figure 1, pursued four basic goals. In the interim, factors such as increasing the capacity of citizenship participation and the institutional capacity of society in disasters have not been considered adequately (Ghafory-Ashtiany & Hosseini 2008).

Existing experience has indicated that after a disaster, urban management does not have the necessary effectiveness. Disasters are growing in domain and impact as a result of the combination of increasing population density and asset stocks, inappropriate and exploitative land use, unplanned settlements and lack of public awareness on risk reduction by authorities and citizens at large (ISDR 2008). One of the issues that causes this is low community participation capacity. Buein Zahra, as a small-sized city in Iran, has still not been provided with the primary components of earthquake risk management. Accordingly, the main purpose of this research was identification of the primary components of earthquake risk management in small-sized cities in Iran and then investigation of their effects on strengthening and improving community participation capacity. Among the small cities in Iran, Buein Zahra was selected. This city, located south of Qazvin Province in the central plateau of Iran, has 18 000 inhabitants. This county is located on the Aipak and Eshtehard faults. It should be noted that the selected region is one of the high-seismic activity cities in Iran, which has witnessed two devastating earthquakes, one of magnitude 7.2 in 1962 and one of 6.3 in 2003. Moreover, in recent years many weak earthquakes have been reported.

This article consists of four parts. Firstly, the primary components of earthquake risk management are investigated. Secondly, the research methodology is explained. Thirdly, the primary components of disaster risk management (DRM) are assessed and their effects on strengthening community participation in Buein Zahra are analysed. Finally, a conceptual model and suggestions for improving participation are presented.

## Building primary components of disaster risk management from the Hyogo Framework for Action

Risk is the probability of damaging events that is derived from the confrontation of risks, social vulnerability and nature (Smith 2013). The goal of risk reduction is to design and create a context for reducing human losses and protecting assets against natural hazards (see also Blaikie et al. 2014; Dowrick 2009). Therefore DRM comprises the range of activities before, during and after a disaster, undertaken to minimise vulnerabilities and disaster risk throughout society, to avoid or to limit the adverse impact of hazards, within the broad context of sustainable development and paying attention to dimensions such as participation, strengthening synergies and knowledge, empowering and increasing capacities, improving the physical environment and its capability (ISDR 2005; Kohler, Julich & Bloemertz 2004; Thomalla et al. 2006).

The Hyogo Framework provides comprehensive action-oriented policy guidance based on a comprehensive understanding of disaster risks that arise from human vulnerability to natural hazards. In the preparatory negotiations on the framework, states stressed the need for specific means, including indicators, to measure progress toward the reduction of disaster risks. In particular, it was requested in Paragraph 33c that the International Strategy for Disaster Reduction (ISDR) system, supported by the United Nations Office for Disaster Risk Reduction (UNISDR) secretariat, coordinate the development of ‘generic, realistic and measurable indicators’ for DRR. It encouraged states to develop and refine such indicators for national use. Indicators, benchmarks and targets are commonly accepted tools to focus and guide development investments, the Millennium Development Goals being an important example.^1^

**FIGURE 2:**
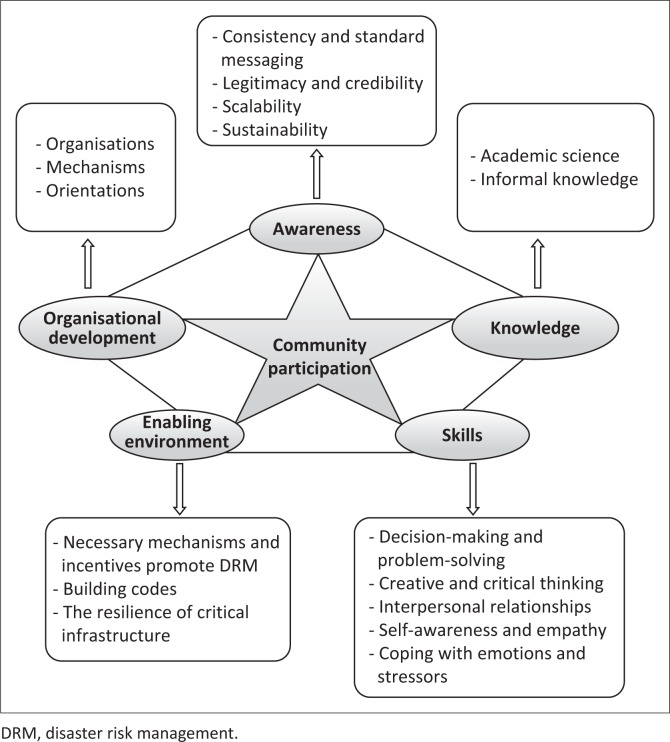
Participation and risk management cycle.

Finally, in the second and third principles, knowledge and awareness were emphasised and the skill component was considered as a subset of individual capacity building. Figure 2 shows the relationship between participation and the components of earthquake risk management.

### Knowledge

Knowledge is created by accumulating and organising information with respect to breadth, depth and amount. Information is ‘data with meaning’ that makes a difference and facts, data and information are necessary mediums for eliciting and constructing knowledge (Weichselgartner & Pigeon 2015).

### Public awareness

Even when academic and practitioner content is freely accessible, it often remains empirical, unstructured and meaningless facts. As a result, although risk information is being generated and disseminated on a large scale, we do not know how far it reaches and whether it changes risk perceptions and public awareness levels (Weichselgartner & Pigeon 2015). The ISDR defines public awareness as the processes of informing the general population, increasing levels of consciousness about risks and how people can act to reduce their exposure to hazards. This is particularly important for public officials in fulfilling their responsibilities to save lives and property in the event of a disaster. Public awareness activities foster changes in behaviour, leading towards a culture of risk reduction. This involves public information, dissemination, education, radio or television broadcasts, use of printed media, as well as the establishment of information centres and networks and community and participation actions (UNISDR 2016).

### Skills

This level relates to the skills, experience and knowledge of people that allow them to perform (Prevention Web 2017). Therefore, over the years, the United Nations Development Programme (UNDP) has invested heavily in training and skill-building of individual capacity in DRR and recovery. However, training is only one methodology for capacity development and it cannot be conducted as an isolated intervention. The ISDR defines ‘capacity-building’ as efforts aimed at developing human skills or societal infrastructures within a community or organisation needed to reduce the level of risk (UNISDR 2017). Skills include capabilities and abilities that are mostly interactive and technical in nature and that empower people in different situations, especially in emergency and crisis situations, for admission and survival. In addition, having the skills for constructive communication and gaining acceptance by others is essential (Hollinger 1987).

### Enabling environment

Sometimes referred to as the ‘societal’ or ‘institutional’ level, capacities at the level of an enabling environment (EE) relate to the broader system within which individuals and organisations function (Wignaraja 2009). Understanding the EE can be obtained from the ‘institutional analyses’, ‘power analysis’ or ‘drivers of change analysis’ increasingly being undertaken by donor organisations as the basis for country assistance plans (Brinkerhoff & Morgan 2010). Capacities at this level relate to all the rules, laws and legislation, policies, power relations and social norms (a set of previously established rules, regulations, procedures and existing conditions) (Wignaraja 2009). They are preserved as restoration levels of its essential basic structures and functions (UNISDR 2017) and they take mitigating actions consistent with achieving that level of protection (Thywissen 2006). To achieve this, based on the action priorities of ISDR (2016), local actions required in order to establish an EE can be identified as shown in Table 1.

**TABLE 1:** Priorities for actions of the International Strategy for Disaster Reduction (2016).

Priorities	Actions
Necessary mechanisms and incentives promote disaster risk management	- Land use and urban planning guideline including urban planning, land degradation and informal and non-permanent housing, access to basic healthcare services
Building codes	- Standardisation of building materials; retrofitting and rebuilding; rehabilitation and reconstruction practices
The resilience of critical infrastructure	- Water, transportation and telecommunications infrastructure, educational facilities, hospitals and other health facilities - Development of early warning systems

*Source*: ISDR, 2016, *Sendai Framework for Disaster Risk Reduction 2015–2030*, viewed 05 March 2018, from https://www.unisdr.org/we/coordinate/hfa-post2015.

### Organisational development and political commitments

The organisational level of capacity comprises the internal policies, arrangements, procedures and frameworks that allow an organisation to operate and deliver on its mandate and that enable the coming together of individual capacities to work together and achieve goals. An EE pertains to the broader system within which individuals and organisations function and that facilitates or hampers their existence and performance. This system comprises institutions (CaDRI 2011). According to North (1991), these are made up of formal constraints (rules, laws and constitutions), informal constraints (norms of behaviour, conventions and self-imposed codes of conduct) and their enforcement characteristics. The development trends and dynamics and the policy environment in which an entity operates in an EE will be assessed, as well as its internal procedures and frameworks on an organisational level.

For effective compliance as well as for sustainability characteristics such as justice or participation, the objective of DRR must be complemented by core organisational objectives (Spangenberg 2002; Spangenberg, Pfahl & Deller 2002). To manage disaster risk, a clear vision, plans, competence, guidance and coordination within and across sectors, as well as participation of relevant stakeholders, are needed. Disaster risk governance fosters collaboration and partnership across mechanisms and institutions for the implementation of instruments relevant to DRR (UNISDR 2015). The CDS’s (2007) set of sustainability indicators was the first to explicitly take into account the institutional dimension of sustainability. In order to measure the effectiveness of the relevant institutions, Spangenberg (2002) analysed regarding Agenda 21 institutional content in three parts, including organisations, mechanisms and orientations. Very broadly defined, political institutions, as analysed by political science, are the rules by which political decision-making and implementation is structured. They can refer to social entities as actors as well as to systems of rules shaping their behaviour, including the mechanisms for rule enforcement. Political organisations encompass both: they are social entities appearing as actors in political processes, as well as systems of rules, structuring political behaviour and facilitating societal orientations. Accordingly, based on the Sendai Framework for Disaster Risk Reduction (UNISDR 2015) priorities, institutional development is analysed as follows (Table 2).

**TABLE 2:** Institutional development characteristics mainstream and integrate disaster risk reduction within and across all sectors.

Characteristics	Features
Organisations	To allocate the necessary resources, including finance and logistics, as appropriate, at all levels of administration for the development and implementation of disaster risk reduction strategies, policies, plans, laws and regulations in all relevant sectors
	To support the role of public service workers to establish or strengthen coordination and funding mechanisms and procedures for relief assistance and plan and prepare for post-disaster recovery and reconstruction
	To strengthen the capacity of local authorities to evacuate persons living in disaster-prone areas
Mechanisms	To carry out an assessment of the technical, financial and administrative disaster risk management capacity
	To formulate public policies, where applicable, aimed at addressing the issues of prevention or relocation, where possible, of human settlements in disaster prone zones, subject to national law and legal systems
	To ensure the continuity of operations and planning, including social and economic recovery and the provision of basic services in the post-disaster phase
	To establish a mechanism of case registry and a database of mortality caused by disaster in order to improve the prevention of morbidity and mortality
Orientations	Promote local frameworks of laws, regulations and public policies that, by defining roles and responsibilities, guide the public and private sectors
	To promote public scrutiny and encourage institutional debates
	To promote the integration of disaster risk reduction considerations and measures in financial and fiscal instruments
	To enhance recovery schemes to provide psychosocial support and mental health services for all people in need

*Source*: Adapted from UNISDR, 2015, *The Sendai Framework for Disaster Risk Reduction 2015–2030*, Resolution A/Res/69/283, viewed 18 March 2017, from http://www.unisdr.org/files/resolutions/N1516716.pdf.

### Community participation

‘Community’ is understood as:

a group of people that may or may not live within the same area, village or neighborhood, share a similar culture, habits and resources. Communities are groups of people also exposed to the same threats and risks such as disease, political and economic issues and natural disasters. (IFRC 2014)

Disaster risk reduction requires engagement and partnership by all of society. It also requires empowerment and inclusive, accessible and non-discriminatory participation, paying special attention to people disproportionately affected by disasters, especially the poorest. Gender, age, disability and cultural perspectives should be integrated into all policies and practices, and women and youth leadership should be promoted. In this context, special attention should be paid to the improvement of organised voluntary work of citizens (UNISDR 2015).

Among the measures to be considered for the achievement of this goal are the choice of the most appropriate participatory system depending on the context, an inclusive approach, the adoption of procedural guarantees and the promotion of DRR among the population including through training and education (Pietropaolo 2015).

These factors can be translated as the basic components of DRR. Neglect of the examined aspects has indeed undermined, in a plurality of contexts, legislative and policy efforts to provide for effective community engagement in such a way as to impair the ultimate goal of building resilience. Table 3 lists the important factors for effective community participation (Pietropaolo 2015).

**TABLE 3:** Important factors in the effectiveness of community participation.

Challenge	Good practices
Tokenistic participatory systems	Even though some advantages can be identified in establishing institutionalised mechanisms, depending on the context, autonomous systems of community consultation might offer a more solid basis for community involvement
Incomplete assessments	All-inclusive approach Possibility to co-opt experts for specific sessions Entrusting each group with a specific field of analysis Separation of women and men during consultations when necessary Engaging community leaders as members and not chiefs of the DRR process
Procedural exclusion	Ensuring participation throughout all the phases of the DRR process and not just the final stages Clearly shaping and communicating the community’s tasks and powers in the DRR process Informing community members of the reasons for not adopting their suggestions
Community exclusion because of lack of interest or capacities	Engaging local media Organising events to sensitise on DRR Engaging community leaders Promoting volunteerism Organising trainings Promoting university courses on DRR Establishing knowledge management centres to facilitate community access to relevant information

*Source*: Pietropaolo, M.G., 2015, *Observations on strengthening community participation in disaster risk reduction in disaster law and policy*, IFRC Publication, Geneva.

DRR, disaster risk reduction.

## Methodology

This study is part of a voluntary project entitled ‘Creating Community Emergency Response Volunteers and Improving DRM in Buein Zahra’; it concentrated on an area of practice and viewed DRM through the eye of volunteer groups.

Surveying based on the field collection method was used to determine the impact of the basic components of risk management on social participation. The data collection tool was a self-made questionnaire based on the framework ‘Guidance on Measuring the Reduction of Disaster Risks and the Implementation of the Hyogo Framework for Action’, and data collection took place in April and May 2017.

### Research methodology

The survey was designed to evaluate how residents perceive their DRM at local level and what factors actually influence their community participation. This survey questionnaire, which contained 113 questions, asked about the sense of satisfaction and level of dedicated and adequate resources DRM. It was completed by approximately 480 participants from Buein Zahra.

### Statistical population

The statistical population is all the citizens of the city of Buein Zahra, which is 18 310 according to the figures calculated in coordination with the Buein Zahra city council and municipality under a voluntary project entitled ‘Creating Community Emergency Response Volunteers and Improving DRM in Buein Zahra’.

Because the main objective of the project was to investigate how to improve public participation in DRM from the viewpoint of voluntary groups, non-probability sampling (voluntary sampling) was used (Vehovar, Toepoel & Steinmetz 2016). Accordingly, the sample was made up of people who self-selected to participate in the survey and had a strong interest in creating community emergency response volunteers. People were informed of the study via billboard and SMS during April and May 2017 and all volunteers were invited to visit the Culture and Islamic Guidance venue of Buein Zahra on 24 May 2017.

On the day of the gathering, firstly the importance of creating community emergency response volunteers was described, and efforts were made to ensure that contributors answered the questions carefully. The volunteers also used the guidance of the research group in the hall to answer questions in case of ambiguity.

An interesting aspect of this survey was the presence of different classes and ages during the gathering. The volunteer sample distribution is shown in Table 4 based on age, sex and education.

**TABLE 4:** Sample community information based on age, sex and education.

Variable	Sample (%)
**Sex**	1-1-1
Female	52.21
Male	47.79
**Age**	2-1-1
<65	2.91
65–45	30.0
44–25	36.87
24–15	26.87
≤14	3.55
**Education**	3-1-1
Illiterate	0.62
Primary	4.38
High school	7.08
Diploma	32.29
Associate’s degree	16.04
Bachelor’s degree	22.3
Master’s degree	17.29

### Research tools

Although the Hyogo Framework provides action guidance, based on a comprehensive understanding of disaster risks, there is still a need for specific tools to measure these indicators. For this purpose, governments are encouraged to develop indicators, benchmarks and targets to measure at national to local levels. The study Indicators of Progress: Guidance on Measuring the Reduction of Disaster Risks and the Implementation of the Hyogo Framework for Action was launched by UNISDR in 2008 for this purpose. However, while the indicators for the Strategic Goals of the Hyogo Framework focus solely on national-level actions, the indicators for the Priorities for Action can be formulated for local and regional levels as well.

Based on this, a set of suggested indicators were proposed to achieve the Hyogo Framework’s five priorities for action. National and local organisations are encouraged to actively use these indicators, in accordance with their mandated areas.

‘Indicators’ are defined here as an explicit measure of an important factor relevant to the subject of disaster risk and its reduction, where the indicator can be used to monitor changes in the status of that factor.

Many of the important factors for which indicators are required will be rather qualitative. Consider the potential indicator ‘Dedicated and adequate resources are available to implement disaster risk reduction plans at all administrative levels’. Its value can only be ‘yes’ or ‘no’, but either of these answers could be misleading, because for example a country with 95% compliance would still need to report ‘no’. One way to address this problem is to qualitatively assess the indicator using a graduated five-point scale from ‘no/minor progress’ through to ‘full/substantial achievement’. Table 5 provides a generic scale of five achievement levels and is proposed as an assessment tool for measuring indicators. The table also includes examples of the application of the assessment tool to the possible indicator ‘A strategy for data provision for disaster risk reduction is in place’. In addition, an indicative table of criteria to illustrate the qualification of achievement for each of the five levels of progress in ISDR (2008) is presented (Table 5). (See UNISDR 2008, annex 5.)

**TABLE 5:** Five-level assessment tool for use in grading achievement of qualitative factors in indicators.

Level	Generic description of achievement	Examples of an assessment of the indicator ‘A strategy for data provision for disaster risk reduction is in place’
5	Comprehensive achievement has been attained, with the commitment and capacity to sustain efforts at all levels.	Systematic, properly resourced processes for data collection and dissemination are in place, with evaluation, analysis and improvements being routinely undertaken. Plans and commitments are publicised and the work is well integrated into other programmers.
4	Substantial achievement has been attained, but with some recognised deficiencies in commitment, financial resources or operational capacities.	Processes for data collection and dissemination are in place for all hazards and most vulnerability factors, but there are shortcomings in dissemination and analysis that are being addressed.
3	There is some commitment and capacity to achieving DRR but progress is not substantial.	There is a systematic commitment to collecting and archiving hazard data, but little awareness of data needs for determining vulnerability factors, and a lack of systematic planning and operational skills.
2	Achievements have been made but are relatively small or incomplete, and while improvements are planned the commitment and capacities are limited.	Some data collection and analysis has been done in the past, but in an ad hoc way. There are plans to improve data activities, but resources and capacities are very limited.
1	Achievements are minor and there are few signs of planning or forward action to improve the situation.	There is little awareness of the need to systematically collect and analyse data related to disaster events and climatic risks.

*Source*: ISDR, 2008, *Indicators of progress: Guidance on measuring the reduction of disaster risks and the implementation of the Hyogo Framework for Action*, United Nations secretariat of the International Strategy for Disaster Reduction (UN/ISDR), Geneva, Switzerland.

DRR, disaster risk reduction.

The indicators listed in Table 5 address the foundations of an effective and well-integrated national DRR programme oriented to implementing the Hyogo Framework. Many other indicators could be formulated, for example to track particular issues of concern, such as the status of vulnerable groups or community emergency response volunteers, sensitive ecosystems or settlements, or particular policy objectives, in which case more detailed indicators are likely to be necessary to adequately assess the desired achievements. Local authorities are encouraged to explore options for identifying and applying relevant and ‘additional’ indicators in areas of concern. The intention at the national and subregional levels will be to develop indicators tailored to specific DRR and recovery projects, programmes and policies. If the data resources are readily available, an indicator may be simple to establish. Subject areas for additional indicators might include the Millennium Development Goals, climate change, governance, corruption, gender equality and other specific development issues related to risk reduction.

In the present study, the only added index to the proposed indicators was the skill index, which, according to the World Health Organization’s guide, became the operational definition and was added with a five-point Likert scale. Table 6 displays the indicators, subindicators, number of items and Cronbach’s alpha.

**TABLE 6:** Criteria and basic indicators of earthquake risk reduction.

Indicators	Subindicators	N	í†
Knowledge	Academia’s best contribution to problem resolution	12	0.74‡
	Informal knowledge		
Public awareness	Consistency and standard messaging	14	0.78
	Legitimacy and credibility		
	Scalability		
	Sustainability		
Skills	Decision-making and problem-solving	22	0.83
	Creative thinking and critical thinking		
	Communication and interpersonal relationships		
	Self-awareness and empathy		
	Coping with emotions and stressors		
Enabling environment	Necessary mechanisms and incentives promote disaster risk management	24	0.75
	Building codes		
	The resilience of critical infrastructure		
Organisational development	Organisations	18	0.76
	Mechanisms		
	Orientations		
Community participation	Tokenistic participation systems	25	0.81
	Incomplete assessments		
	Procedural exclusion		
	Community exclusion because of a lack of interest or capacity		

*N*, number of items.

† , Cronbach’s *α*.

‡ , 0.7 ≤ *α* < 0.8 – acceptable.

### Ethical considerations

This article followed all ethical standards for research without direct contact with human or animal subjects.

## Measure of disaster risk management in Buein Zahra

A descriptive analysis of the primary components of the DRM items is shown in Tables 7 and 8. Table 7 shows the status of each item in Buein Zahra at three levels: low, medium and high. Table 8 also shows the significant means of each indicator.

**TABLE 7:** Cluster classification of the basic components of disaster risk management.

Indicators		Cluster classification of component levels†		
		Low	Medium	High‡
**Knowledge**		24.74	36.81	38.45
	Formal knowledge	26.30	35.54	38.16
	Informal knowledge	23.17	38.09	38.74
**Awareness**		21.85	31.64	46.51
	Consistency and standard messaging	19.10	31.54	49.36
	Scalability	12.13	35.99	51.88
	Legitimacy and credibility	32.22	28.57	39.21
	Sustainability	23.95	30.45	45.60
**Skills**		40.66	33.64	25.70
	Decision-making and problem-solving	42.82	37.54	19.64
	Creative thinking and critical thinking	41.39	33.76	24.85
	Interpersonal relationships	27.59	32.97	39.44
	Self-awareness and empathy	46.26	29.03	24.71
	Coping with emotions and stressors	45.24	34.91	19.85
**Community participation**		44.99	26.99	28.02
	Tokenistic participatory systems	38.75	30.10	31.15
	Incomplete assessments	41.05	28.97	29.98
	Procedural exclusion	52.75	24.01	23.24
	Community exclusion	47.42	24.87	27.71
**Enabling environment**		37.95	36.49	25.56
	Necessary mechanisms and incentives	37.35	37.51	25.14
	Building codes	32.11	36.01	31.88
	The resilience of critical infrastructure	44.39	35.96	19.65
**Organisational development**		44.16	33.30	22.54
	Organisations	44.01	34.12	21.87
	Mechanisms	48.93	32.54	18.53
	Orientations	39.55	33.23	27.22

† , distribution percentage of responses.

‡ , sum total is equal to 100.

**TABLE 8:** Respondents’ disaster risk management components.

Indicators	*N*	Mean	Item average	Mean difference	*t*	p*
Knowledge	480	43.5	30.0	+ 7.90	14.70	0.00
Awareness	480	54.8	35.0	+ 13.60	33.25	0.00
Skills	480	41.3	55.0	−8.10	−15.03	0.00
Community participation	480	47.3	62.5	−9.60	−22.69	0.00
Enabling environment	480	49.3	60.0	−5.68	−10.69	0.01
Organisation development	480	28.4	45.0	−10.90	−26.17	0.00

*N*, number; *t, t*-statistic.

* , *p* < 0.1.

### Public awareness

According to Table 7, public awareness is promising. The high level of public awareness maybe partly because of the past experience of earthquakes and the importance of the issue for the volunteers participating in the research. About 46% were at an acceptable level, about 32% were on average and 22% were low. Among the subindicators of public awareness, scalability, consistency and standard messaging, sustainability, legitimacy and credibility were higher than average.

Now, with the advancement of various media, it seems that a small city such as Buein Zahra is well covered, and improving the sustainability and legitimacy of the state-owned media can lead to more effective communication.

### Knowledge

The knowledge component indicates that about 38% of the population samples stated that the related academic and non-academic knowledge was provided and researches and reports about earthquake and fault activity were available. The research reports present a detailed assessment of the current situation in different places. In this indicator, academic research is in a better position than non-academic research.

### Skills

The 22 skill indicators showed a low level among the volunteer citizens, so that about 75% of society had moderately lower skills.

Among the subindicators, effective communication and interpersonal relationship skills were in a better situation, but the rest did not show an acceptable status.

### Community participation

The situation of social participation suggests that about 70% of the sample population believed that the necessary grounds for social participation were not provided. Among the subindicators, procedural exclusion, community exclusion, incomplete assessments and tokenistic participatory systems had the lowest averages.

### Enabling environment

The 24 indicators of EE showed a medium downward situation, where the subindicator ‘resilience of critical infrastructure’ had a less acceptable level and the building codes had an acceptable average status.

### Organisational development

The results presented in Table 7 indicate that about 76% of the population assessed the existing mechanisms, orientations and organisational structure in dealing with earthquake crisis as ineffective. Meanwhile, mechanisms have the lowest mean and organisations and orientations the next lowest.

In addition, based on Table 8, the results of the *t*-test indicate that there is a significant difference between basic DRM indicators and their item means. This difference is significant at alpha level of 0.05, indicating that among the components of DRM, the average of public awareness is higher.

### Effective mechanisms of disaster risk management through public participation

By using multivariate linear regression analysis, the linear combination of the relationship between independent variables and dependent variable can be predicted. Although in this way the direct impact of each variable could be predicted, the indirect effects, the conceptual and theoretical model of research has not been formed.

**FIGURE 3:**
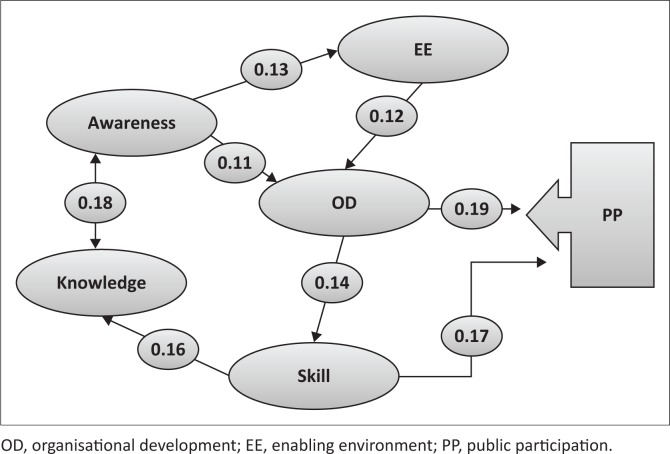
Empirical model of factors affecting participation in disaster risk management.

Accordingly, the path analysis method was used. The path analysis, first developed by Wright (1934), is an extension of the multivariate regression method to generate causal models. In the path analysis, the arrows determine the causal effects of variables towards the intermediate and final variables and how to affect direct and indirect effects and, finally, the theoretical model, after the test run is converted to the experimental model of research (MacKinnon 2012; Suhr 2008). The matrix of correlation coefficients and effects of variables are shown in Tables 9 and 10. In addition, Figure 3, based on the standardised regression coefficients of DRM, displays the effective factors of public participation.

**TABLE 9:** Correlation coefficients of disaster risk management variables.†

Variable	PP	Awareness	Skill	OD	Knowledge	EE
Awareness	0.16	-	-	-	-	-
Skill	0.19	0.05	-	-	-	-
OD	0.23	0.14	0.17	-	-	-
Knowledge	0.04	0.22	0.19	0.15	-	-
EE	0.18	0.16	0.13	0.10	0.14	-

OD, organisational development; EE, enabling environment; PP, public participation.

† , Matrix of correlation coefficients of variables.

**TABLE 10:** The effects of disaster risk management variables on public participation.

Variables	Direct effect	Indirect effect	Non-causal effect	Total causal effect	*R*^2^†
Awareness	0.00	0.026	0.130	0.026	0.16
Skill	0.17	0.004	0.016	0.174	0.19
OD	0.19	0.020	0.020	0.210	0.23
Knowledge	0.00	0.005	0.035	0.005	0.04
EE	0.00	0.026	0.154	0.026	0.18

OD, organisational development; EE, enabling environment.

† , *R*^2^ denotes the coefficient of determination.

The different types of impact on public participation were calculated as follows:

- **Direct impact:** A regression coefficient of each variable on participation. It can be obtained from the output of the regression analysis.
- **Indirect impact:** Firstly all paths of the indirect effects of each independent variable on the dependent variable must be multiplied and then all these effects are summed up.
- **Total impact:** The sum of the direct and indirect effects of each variable.
- **Variables that have only a direct impact on participation**: Two variables – organisational development (OD) and skill – have only a direct impact on participation as an intermediate dependent variable. The effect of each of these variables on participation is as follows:
  ■ The direct impact of the OD is equal to 0.19, which indicates that for each unit of change in its value, the participation rate will change by 0.19 units.
  ■ The skill variable also has a direct impact on participation of 0.17.
- **Variables that have only indirect impact on participation**: three variables – awareness, knowledge and enabling environment (EE) – are variables that have only indirect impact by intermediate variables. The effect of each of these variables on participation is as follows:
  ■ Awareness affected participation through three paths: firstly through OD (0.11); secondly through EE and then OD; thirdly through EE, OD and then skill. With regard to the direction of the path coefficient, these effects are incremental, that is, with the increase of awareness, the participation rate will also increase.
  ■ Knowledge also affected participation through three paths: firstly through awareness, EE and OD; secondly through awareness, EE, OD and skill; thirdly through awareness and OD.
  ■ In an EE, two paths are recognisable: one through OD and the other through OD and skill.
- **Variables that encompass both direct and indirect impacts on participation:**
  ■ The skill variable has both direct and indirect effects. For indirect effects, two directions (A and B) can be considered. In Direction A, the skill variable has an impact on participation through knowledge, awareness, EE and OD. In Direction B, through knowledge, awareness and OD, a path is recognisable.
  ■ Organisational development has an impact on public participation, both directly with the coefficient 0.19 and indirectly through the skill variable with path coefficient 0.14.
  ■ In sum, based on the results of the total impact coefficient, we can say that OD, skill, awareness, EE and knowledge have the most impact on public participation in Buein Zahra. Also according to the empirical model, the variables OD and skill were found to be middle variables and the variables awareness, EE and knowledge were detected as external variables.

Figure 3 shows the correlation coefficient in path analyses and how to change the values of variables by increasing or decreasing the compared variables. Using this method and calculating the coefficient of correlation between external variables, it can be said that the selection of variables was not a mosaic but there were interactions between them, and the variables were selected according to the theoretical model.

## Discussion and conclusion

Today, the DRM strategies in Iran are growing. As part of this change, centralised planning is being replaced with community-based planning. The reason is that with a state-oriented view and centralised planning, the elaboration and implementation of strategies occurs in a top-down manner; it imposes many costs on the state and is not sufficiently effective. However, in the community-based orientation, people have a significant role and influence at different stages of disaster management. Moreover, building resilient communities involves ensuring that communities and community members have there sources, capacities and capabilities necessary to bounce back and recover in a manner that minimises disruption and facilitates growth (Paton & Johnston 2001). The results show that the awareness and knowledge levels of Buein Zahra have been improved, but there is still no basis for creating community-based planning because these variables improve public participation through other variables. Currently, it seems that neither public awareness nor knowledge has come to be understood as a skill, nor has necessary OD taken place. Just raising public awareness, education and knowledge without creating the necessary ground for OD and skills will not create community-based disaster management in Iran. Accordingly, the most important factors for moving toward this goal are organisational development and then skill improvement. Knowledge and public awareness, which strengthen skills and organisational development, over time can lead to real participation. Therefore, after natural disasters such as earthquakes, local people cannot participate collectively in coping with them, and we see private and family participation at a limited level in the aftermath of the disaster. Moreover, in order to make it a practical reality, crisis and disaster mitigation requires not only the participation of the individual within the vulnerable community but also the involvement of related institutions, NGOs and the general public (Chen, Liu & Chan 2006; Newport & Jawahar 2003).

For organisational development, in line with Pietropaolo (2015), two main challenges are identifiable: the first challenge is related to decentralisation of processes. The necessary orientations are still needed to promote public rules and policies that define the roles and responsibilities of the public and private sectors. Local capacity to organise is not strengthened, and the rules for the assignment of affairs are not laid down or are not fully implemented.

Secondly, some functional mechanisms and social participation procedures in risk management have been predicted, such as environmental NGOs or earthquake and safety manoeuvres, but they only show levels of apparent features of participation, representing symbolic participation, which does not have an impact on real public participation during the disaster. Strengthening organisational development, as a platform for local collaborative activities, requires the political will to delegate some responsibilities to civil institutions and policies to facilitate the activities of volunteer groups in various social areas. The adoption of participatory policies and its related political will and reinforcement of skills, with regards to suitable knowledge and awareness, can help to ensure public participation during a disaster.

## Footnotes

1 Strategies linked to ISDR for moving ahead on this goal were outlined in the ‘Road Map towards the Implementation of the United Nations Millennium Declaration’ (UN 2001).
